# Interdisciplinary insights on intubation checklist use in the ICU: a qualitative study

**DOI:** 10.1093/atsscholar/aapag021

**Published:** 2026-04-22

**Authors:** Samuel I Garcia, Philippe R Bauer, Jennifer L Ridgeway, Diana J Kelm

**Affiliations:** Division of Pulmonary, Critical Care and Sleep Medicine, Mayo Clinic College of Medicine and Science, Rochester, MN, United States; Department of Emergency Medicine, Mayo Clinic College of Medicine and Science, Rochester, MN, United States; Division of Pulmonary, Critical Care and Sleep Medicine, Mayo Clinic College of Medicine and Science, Rochester, MN, United States; Robert D. and Patricia E. Kern Center for the Science of Health Care Delivery, Mayo Clinic, Rochester, MN, United States; Division of Pulmonary, Critical Care and Sleep Medicine, Mayo Clinic College of Medicine and Science, Rochester, MN, United States

Airway management in the intensive care unit (ICU) is a life-saving procedure, where first-attempt success (FAS) is essential to reduce complications such as hypoxia, hypotension, cardiac arrest, and death.^1^^,^^2^ However, despite increasing FAS rates, procedural complications remain high.^2^ Many of these adverse events are attributed to preventable errors, human factors, and team-based failures.^3^^,^^4^

While intubation checklists aim to improve safety, they are often physician-driven and may not fully reflect the interdisciplinary nature of emergent intubations. Understanding the perspectives of all team members is essential to inform the development of practices that foster collaboration and reduce team-related failures. Building on prior empirical work that identified checklist use as a valuable safety intervention with substantial opportunities for improvement, this study examined interdisciplinary perceptions of checklist use during emergent intubations to identify strategies to improve team coordination and enhance patient safety.^5^

## Study design and methods

### Settings and participants

We conducted a descriptive qualitative study using focus groups (FGs) of ICU intubation team members from three tertiary care centers: a medical ICU in Minnesota with routine checklist use, a multispecialty ICU in Arizona without a standardized checklist, and a multispecialty ICU in Florida with infrequent checklist use. Sites were selected to capture variation in practice and checklist use. Participants included attending physicians, fellows, advanced practice providers, nurses, and respiratory therapists (RTs). The study was deemed exempt by the Institutional Review Board.

### Interdisciplinary focus group sessions

Participants were recruited via purposive sampling through email to join 60-minute virtual FGs with mixed roles. Participants were selected based on predefined characteristics aligned with the study aims, specifically active clinical roles in airway management, to ensure representation from key professional groups. FGs were conducted using video-conferencing software and moderated by trained team members (SG, DK) not in the participants’ supervisory chains to encourage open discussion. A semi-structured interview guide (Supplementary Appendix 1) developed iteratively from prior work, explored communication, safety, and teamwork, while allowing new ideas to emerge inductively.^5^ Participants received a small remuneration, and confidentiality, voluntary participation, and psychological safety were emphasized.

### Data analysis

Sessions were audio-recorded, transcribed verbatim, and analyzed using a hybrid deductive-inductive approach. Two investigators (SG, DK) applied an a priori, literature-informed coding framework (Supplementary Appendix 2), while allowing novel codes to emerge inductively from the data. Dual coding and iterative codebook refinement ensured analytic rigor. Cross-site comparison and team debriefing facilitated triangulation and resolution of discrepancies. Themes were identified through iterative clustering of related codes, exploring potential convergence and divergence among disciplines and institutions. Dedoose (www.dedoose.com) qualitative software facilitated coding and thematic analysis.

## Results

Between November 2023 and January 2024, 11 interdisciplinary FGs were conducted with 54 participants: 24 nurses (44%), 17 physicians (31%), 7 advanced practitioners (13%), and 6 RTs (11%). The baseline demographics of participants are provided in Table 1.

**Table 1:** Baseline demographics of interdisciplinary focus group session participants.

	Minnesota	Florida		Arizona	
**Focus Group Session Characteristics**					
**Number of Sessions**	4		4		3
**Average Session Length, min**	53		46		44
**Participant Demographics**					
** Number of participants**	33		14		7
**Sex, Female (%)**	62		81		83
**Team Members**					
**Physician**	13		3		1
**Advanced Practice Provider**	4		2		1
**Respiratory Therapist**	4		1		1
**Registered Nurse**	12		8		4

Three major themes emerged: (1) Checklist implementation and format; (2) Interprofessional communication; and (3) Clinical context and environment. Themes were consistent across sites and professions, with no major differences in the domains identified. Table 2 summarizes themes by category with exemplary quotes from participants.

**Table 2:** Summary of findings and representative quotations.

Themes	Representative Quotation (s)
**Checklist Implementation and Format**	Advanced Practitioner: “I envision the provider discussing the medication plan with nursing along with the elements that nursing feel is important. Then, there would be another box where the provider discusses the airway plan with RT, with the elements that RT thinks are important.”
	Respiratory Therapist: “I think that separating [the checklist] to focus on the multidisciplinary areas would be very helpful. Because there are currently so many items on the checklist that do not relate to our role as much.”
	Nurse: “I think if there’s a way that you could create a box for each discipline for their tasks to read off it might be a better organized intubation checklist.”
	Advanced Practitioner: “The benefit of a checklist is that you can say to a new team member, ‘Okay, here’s our thing. This is the way we do things here. If you have questions, look on the back.’”
	Nurse: “It comes back to the separation of roles. It would be easier to find the steps that you actually are responsible for. A checklist must be driven to the least experienced person in the room.”
	Nurse: “I feel like it would be beneficial to do an intubation simulation and use the checklist to actually practice that before the new nurses hit the floor.”
	Nurse: “The best time [to review the checklist] is after I have drawn up my medications so I can focus on each item. Otherwise, if I am in the middle of getting supplies ready, it’s difficult to focus.”
	Nurse: “I know that we do mock codes in the hospital. I feel like a multidisciplinary simulation [for emergent intubations] would be helpful, where everyone comes together to give feedback.”
**Interprofessional Communication**	Advanced Practitioner: “We need to use closed-loop communication because you may say, “We need wall suction,” and assume somebody is taken responsibility for it, but everybody is just assuming that someone else is responsible for it and then it gets missed.”
	Provider: “It has to be repeated back. I think that close communication for equipment is especially important.”
	Advanced Practitioner: “For the medications, having someone repeat back what they are planning to do, and then call out the medications as they are pushing them is important.”
	Provider: “Closed-loop [communication] is always important, so I think incorporating that in the final checklist is very helpful.”
	Respiratory Therapist: “It would be helpful for our new staff to go through and say, “What’s our plan if the first line fails? Do we have backup equipment ready?” I think it would be a good education tool.
	Provider: “The multidisciplinary theme is important to optimize communication and teamwork.”
**Clinical Context and Environment**	Provider: “It should be a standardized procedure, and I think the checklist helps reinforce that every time.”
	Advanced Practitioner: “We have a lot of new nurses and APPs. When I was new, I was freaking out and it was really nice to have a clear list of things that I knew I was responsible for”
	Nurse: “As a newer nurse, you don’t know the questions to ask, and it just helps get everything that you need in order faster.”
	Nurse: “It’s good at least to have a standard expectation of foundation, and that’s what checklists are made to do. It’s good for everyone to start off with the same foundation.”
	Advanced Practitioner: “I think a checklist are very good, especially when you have a lot of new team members coming from different areas.”
	Provider: “It’s not uncommon when we do an intubation that there’s at least one or two people in the room who haven’t done very many intubations before.”
	Provider: “[The checklist] helps standardize the practice to keep everyone on the same page.”

### Checklist implementation and format

Effective checklist use requires deliberate interdisciplinary training and routine practice. Participants recommended in-situ interdisciplinary “*mock intubations*” to enhance preparedness and operationalize checklist use in clinical practice.

A concise, one-page checklist organized by procedural stage was preferred, with a more detailed version available on the back to support training and education. Role-specific, color-coded sections (eg, airway operator, RT, nurse) were suggested to clarify responsibilities.

### Interprofessional communication

Structured communication was essential for shared mental models and role clarity. For medications, they noted that the airway operator should state the generic name, dose, and order, and that this should be followed by closed-loop communication from the nurse as the checklist is completed. The airway operator should also outline the primary, backup, and contingency plans with RT confirming during the procedural pause. Linking tasks to specific roles (eg, medications to nursing, equipment to RTs) promotes accountability. Participants recommended incorporating these components into the checklist and completing them during a structured post-preparation time-out involving all team members to promote shared situational awareness.

### Clinical context and environment

Emergent intubations often involve time pressure, cognitive load, and variable team experience, increasing error risk. Participants said these factors also contribute to common barriers to routine checklist use, including uncertainty about who should read the checklist, difficulty locating the checklist when needed, limited awareness of its availability in some units, and the absence of a formalized checklist in others. Participants additionally described variability in procedural approaches among team members, highlighting the need for standardization to promote consistent and safe care while allowing for patient-specific adjustments.

Staffing turnover and varying experience levels add to the complexity. A standardized checklist, combined with routine mock intubation training sessions, was identified as a key educational strategy to support new staff, align expectations, and enhance team performance.

## Discussion and conclusion

This study underscores the value of an interdisciplinary approach to airway management and offers strategies for integrating checklists into practice. Key recommendations include incorporating checklists into a dedicated procedural time-out, using standardized interdisciplinary communication, and implementing mock intubation training. Across the studied units, checklist use was variable and airway operator–focused, suggesting a need to explicitly incorporate interdisciplinary roles to enhance collaboration and safety. Our findings align with the TeamSTEPPS framework, emphasizing teamwork, communication, and situational awareness as foundations of team performance.^6^

While FAS is an important benchmark in airway management, preventing procedural complications also requires preparation, appropriate medications, equipment selection, and coordinated teamwork.^2^^,^^4^^,^^7^ Checklists can support these processes but must be deliberately integrated into workflows. Without regular use and team endorsement, even well-designed checklists may be underutilized and ineffective in high-risk settings.^8^^,^^9^

Routine interdisciplinary in-situ mock intubations, adapted from the well-established model of mock codes, as an educational intervention to reinforce checklist practices, may improve team coordination, and enhance procedural safety.^10^ Embedding these practices into onboarding and continuing education was seen as beneficial, especially in settings with variable experience levels and frequent staff turnover. While promising, the optimal frequency, fidelity, structure, and measurable impact of mock intubations on key outcomes like FAS and adverse event reduction remain unknown and warrant further investigation.

In summary, these findings provide insight into a team-centered approach to airway management, supported by interdisciplinary checklist use and reinforced through mock intubation training sessions to strengthen communication, team coordination, and procedural safety.

## Supplementary Material

aapag021_Supplementary_Data

## Notation of prior abstract publication/presentation

Accepted for Oral presentation at CHEST 2024 in Boston, MA.

## Appendix 1. Semi-Structured Focus Group Interview Guide

*Thank you for participating in the study! This study has been funded by the Department of Medicine Catalyst Award and the PCCM Small Grants Award.*

*The goal of this study is to assess the value of an intubation checklist and to identify opportunities to improve team coordination, communication, and procedural safety during emergent endotracheal intubations by incorporating your feedback.*

*There are no right, or wrong answers so feel free to speak freely. This discussion is being recorded and later transcribed for study purposes, but your names will not be recorded thus all your comments will be anonymous.*

**Opening Question** 

- What is the value in using a checklist in preparing for intubations?

**Broad Questions** 

- What are the strengths of using an intubation checklist?
- What are the weaknesses or limitations?
- How could the checklist be improved?
- What elements should be included in an intubation checklist?
- What should be excluded from an intubation checklist?

**Specific Questions** 

- Who should lead or perform the checklist?
- When should it be performed?
- How should it be performed?
- What is the optimal length of the checklist?

**Conclusion** 

- If you could create the ideal intubation checklist, what would that look like and how would it be used?

**Summary** 

- Ask the assistant moderator to quickly summarize the discussion and to ask clarifying questions.
- Closing comments from the group.

## Appendix 2. A Priori Codebook Used in Initial Deductive Coding

A limited set of a priori codes informed by prior literature on airway management, intubation checklists, and interprofessional teamwork was used during initial transcript coding.

**Table:**

Code	Description
**Checklist Utilization**	References to whether and how an airway checklist is used in clinical practice, including timing and consistency during emergent intubations.
**Preparation**	Discussion of procedural planning prior to intubation, including preparation of medications, patient positioning, and team readiness.
**Equipment**	References to preparing airway equipment, monitoring devices, suction, and backup airway tools prior to intubation.
**Roles**	Discussion of assignment of responsibilities among team members during emergent intubation.
**Communication**	Verbal communication among physicians, nurses, and respiratory therapists regarding the airway plan and procedural steps.
**Contingency Planning**	Discussion of backup airway strategies or plans in the event of failed intubation or complications.
**Safety**	Participant perceptions regarding how checklist use influences patient safety or prevention of missed steps.

## Abbreviations:

ED: Emergency Department
ICU: Intensive Care Unit
