# Supplementary material for: Interdisciplinary insights on intubation checklist use in the ICU: a qualitative study
Source: ATS Sch. 2026 Apr 22;7(3):188–91. doi: 10.1093/atsscholar/aapag021 (PMC13519337; doi:10.1093/atsscholar/aapag021)
Supplement: Supplementary file 1 [file aapag021_supplementary_data.zip › aapag021_Supplementary_Data/Appendix file.pdf]

*ATS Scholar*, 2026, 7(3), 1
https://doi.org/10.1093/atsscholar/aapag021
Published: April 22, 2026
Brief Reports

# Appendix 1. Semi-Structured Focus Group Interview Guide

*Thank you for participating in the study! This study has been funded by the Department of Medicine Catalyst Award and the PCCM Small Grants Award.*

*The goal of this study is to assess the value of an intubation checklist and to identify opportunities to improve team coordination, communication, and procedural safety during emergent endotracheal intubations by incorporating your feedback.*

*There are no right, or wrong answers so feel free to speak freely. This discussion is being recorded and later transcribed for study purposes, but your names will not be recorded thus all your comments will be anonymous.*

**Opening Question**

- What is the value in using a checklist in preparing for intubations?

**Broad Questions**

- What are the strengths of using an intubation checklist?
- What are the weaknesses or limitations?
- How could the checklist be improved?
- What elements should be included in an intubation checklist?
- What should be excluded from an intubation checklist?

**Specific Questions**

- Who should lead or perform the checklist?
- When should it be performed?
- How should it be performed?
- What is the optimal length of the checklist?

**Conclusion**

- If you could create the ideal intubation checklist, what would that look like and how would it be used?

**Summary**

- Ask the assistant moderator to quickly summarize the discussion and to ask clarifying questions.
- Closing comments from the group.

# Appendix 2. A Priori Codebook Used in Initial Deductive Coding

A limited set of a priori codes informed by prior literature on airway management, intubation checklists, and interprofessional teamwork was used during initial transcript coding.

| Code | Description |
|---|---|
| **Checklist Utilization** | References to whether and how an airway checklist is used in clinical practice, including timing and consistency during emergent intubations. |
| **Preparation** | Discussion of procedural planning prior to intubation, including preparation of medications, patient positioning, and team readiness. |
| **Equipment** | References to preparing airway equipment, monitoring devices, suction, and backup airway tools prior to intubation. |
| **Roles** | Discussion of assignment of responsibilities among team members during emergent intubation. |
| **Communication** | Verbal communication among physicians, nurses, and respiratory therapists regarding the airway plan and procedural steps. |
| **Contingency Planning** | Discussion of backup airway strategies or plans in the event of failed intubation or complications. |
| **Safety** | Participant perceptions regarding how checklist use influences patient safety or prevention of missed steps. |

**Received:** July 1, 2025. **Accepted:** March 26, 2026
© The Author(s) 2026. Published by Oxford University Press on behalf of the American Thoracic Society.
This is an Open Access article distributed under the terms of the Creative Commons Attribution License (https://creativecommons.org/licenses/by/4.0/), which permits unrestricted reuse, distribution, and reproduction in any medium, provided the original work is properly cited.
